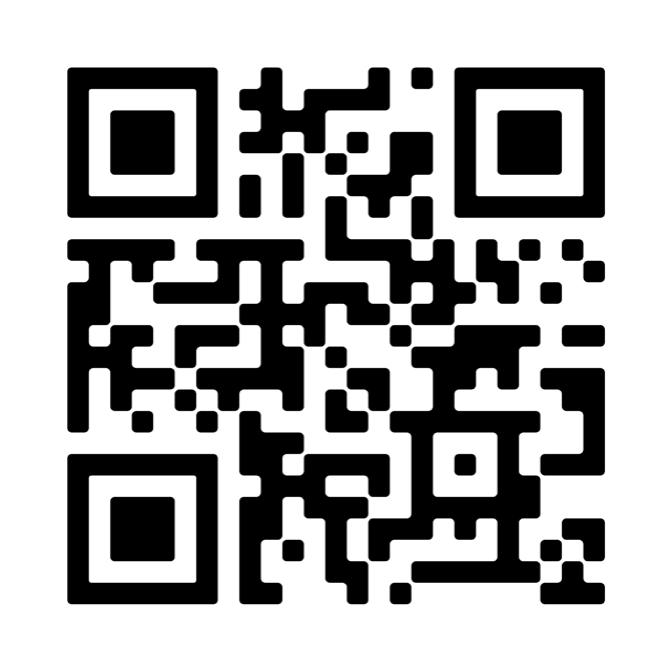# Intravesical misplacement of vaginal contraceptive ring: a video report and review of the literature

**DOI:** 10.52054/FVVO.16.2.016

**Published:** 2024-06-28

**Authors:** G Panico, G Campagna, S Mastrovito, D Arrigo, D Caramazza, G Scambia, A Ercoli

**Affiliations:** Department of Woman, Child and Public Health, Fondazione Policlinico Universitario A. Gemelli IRCCS, 00168 Rome, Italy; Precision Gynaecological Surgery Unit, Dipartimento Centro di Eccellenza Donna e Bambino Nascente, Fatebenefratelli Gemelli Isola Tiberina, 00186 Rome, Italy; Institute of Obstetrics and Gynecology, Università Cattolica del Sacro Cuore, 00168 Rome, Italy; Unit of Gynecology and Obstetrics, Department of Human Pathology of Adults and Developmental Age, University Hospital “G. Martino”, 98100 Messina, Italy

**Keywords:** NuvaRing®, contraceptive vaginal ring, intravesical foreign body, cystoscopy

## Abstract

**Background:**

The NuvaRing®, a hormonal vaginal contraceptive device, has gained widespread usage due to its favourable efficacy and safety profiles. Exceedingly rare instances of unintended misplacement in the bladder have been reported. This study presents a review of the literature and the first video report illustrating the extraction of an intravesical NuvaRing®, discussing diagnostic and therapeutic approaches.

**Objective:**

To illustrate an effective method for intravesical NuvaRing® retrieval and raise awareness about this unusual complication.

**Materials and Methods:**

A 27-year-old patient with low urinary tract symptoms related to NuvaRing® misplacement underwent diagnostic procedures, including ultrasound and diagnostic cystoscopy. A cystoscopic extraction under general anaesthesia was performed.

**Main Outcome Measures:**

The effectiveness of pelvic ultrasound for diagnosing an intravesical foreign body, successful cystoscopic removal of NuvaRing® from the bladder, and symptom resolution were assessed.

**Results:**

The intravesical NuvaRing® was identified through pelvic ultrasound. During cystoscopy, the ring was detected inside the bladder. Multiple attempts with cystoscopic alligator graspers were made; the NuvaRing® was eventually extracted using transurethral Heiss forceps. The patient experienced minimal blood loss and was discharged the following day, reporting relief from symptoms.

**Conclusions:**

Unintentional NuvaRing® placement in the bladder is an extremely rare event that healthcare providers should consider when patients present with urinary symptoms and pelvic pain. Pelvic ultrasound is an efficient diagnostic tool, possibly averting the need for further imaging techniques. Cystoscopy remains the preferred method for diagnosis and treatment. This video report illustrates an effective technique for NuvaRing ® extraction, especially when appropriate graspers are unavailable. Adequate instruction on NuvaRing® insertion should always be emphasised.

## Learning objective

The primary learning objective is to illustrate a successful method for the retrieval of an intravesical NuvaRing ®, emphasising the importance of considering this rare event when users of vaginal contraceptive ring present with urinary symptoms and/or pelvic pain. Furthermore, our aim is to emphasise the diagnostic efficiency of pelvic ultrasound and underscore the critical importance of providing adequate instruction on NuvaRing® insertion to prevent potential complications.

## Introduction

The NuvaRing® (Organon, Kenilworth, New Jersey) is a hormonal contraceptive method consisting of a nonbiodegradable, latex-free, flexible, and transparent vaginal ring containing 2.7 mg of ethinyl oestradiol (EE) and 11.7 mg of etonogestrel. It has an outer diameter of 54 mm, a cross-sectional diameter of 4 mm, and can easily be compressed to 1 cm or less during vaginal insertion. Typically self-administered by the patient, it is designed to remain in place for a period of 3 weeks, followed by a ring-free interval of 1 week during which withdrawal bleeding takes place.

While most of its potential side effects are related to systemic hormonal factors, local adverse events have also been documented in 2-4% of users. These manifestations encompass sensations of a foreign body, increased rates of leucorrhoea, vaginitis, coital difficulties, and instances of expulsion (Roumen and Mishell, 2012; Wieder and Pattimakiel, 2010).

Of particular concern is the exceedingly rare event of unintentional placement of vaginal rings into the bladder. Since its introduction in 2001, only five cases have been reported in the literature. In each instance, patients presented with obstructive and irritative symptoms including dysuria, urinary frequency, urinary retention with fractionated voiding, incontinence, and haematuria. In most cases, an initial misdiagnosis of lower urinary tract infection led to unsuccessful antibiotic treatments. In reported cases, successful extraction of the ring was achieved through cystoscopy, either in an outpatient setting or with deeper sedation and general anaesthesia, employing different techniques (Baker and Barish, 2014; Bhaduri et al., 2009; Ehdaie et al., 2013; Tarragón Gabarró et al., 2009; Teal and Craven, 2006). In this context, we present the first video report detailing the removal of an intravesical NuvaRing®.

## Patients and methods 

A 27-year-old nulliparous woman presented at our outpatient urogynaecology clinic complaining with storage and voiding urinary symptoms including dysuria, urgency, frequency, and stranguria for over a year. Dysuria was referred to as the most relevant symptom. The patient had neither notable medical, surgical, or psychiatric conditions nor relevant family history.

She reported that symptoms had appeared shortly after insertion of a NuvaRing® contraceptive device 12 months prior, which was inserted without discomfort, without the use of its applicator. After three weeks, the patient was unable to remove the ring and reported its loss, attributing it to spontaneous expulsion, and continued using the NuvaRing® over the following months. She was diagnosed with uncomplicated cystitis and was prescribed broad-spectrum antibiotics with no resolution of symptoms, leading to treatment for recurrent cystitis over the subsequent year.

**Figure 1:**
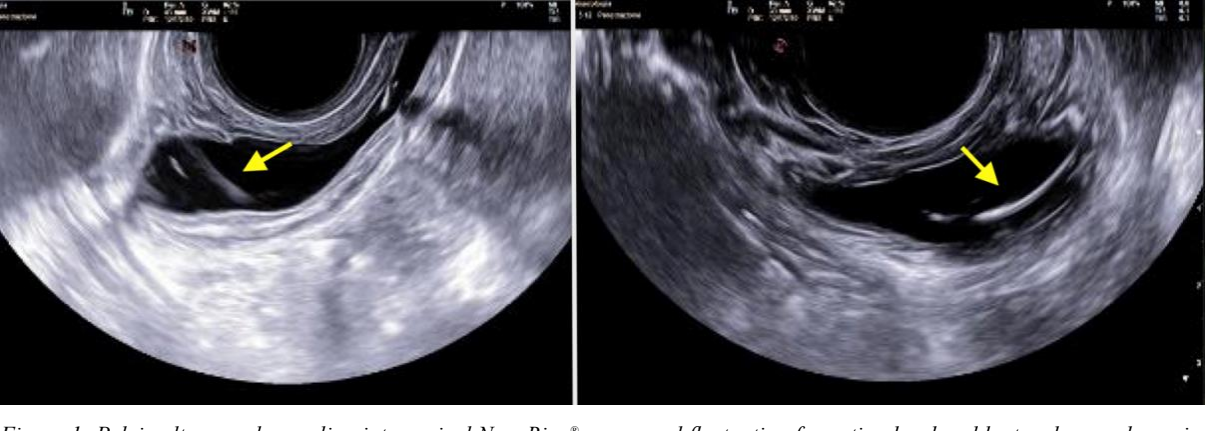
Pelvic ultrasound revealing intravesical NuvaRing® as an oval fluctuating formation bordered by two hyperechogenic, non-vascularized lines.

Physical examination did not show any notable abnormalities. A transabdominal and transvaginal ultrasound was performed, revealing an intravesical oval fluctuating formation bordered by two hyperechogenic, non-vascularized lines, indicative of a foreign body (Figure 1). No additional radiologic exams were necessary.

In an office setting, a diagnostic cystoscopy confirmed the presence of the NuvaRing ®. Subsequently, a cystoscopic extraction procedure was scheduled in the operating room under general anaesthesia, utilising a 23 French rigid cystoscope. The patient was advised about the procedure and signed an informed consent allowing the use of personal data.

## Results

Under general anaesthesia, the patient was positioned in the dorsal lithotomy position with both legs on Allen stirrups with arms along the body. She received antibiotic prophylaxis consisting of 2 g of cefazoline 1 h before surgery.

Intraoperatively, the NuvaRing® was identified floating inside the bladder lumen. Both the urethral and vesical mucosae exhibited no abnormalities, apart from slight hyperaemia. Some infructuous removal attempts were made using an alligator grasper. NuvaRing ® retrieval proved to be challenging due to the limitation of available grasping forceps, which had relatively short branches and inadequate grasping capacity to extract the ring from the inner urethral orifice.

Ultimately, removal was successfully carried out under cystoscopic guidance by introducing Heiss forceps transurethrally, positioned between the upper urethral wall and the cystoscope. This method proved to be safe and effective, overcoming the shortage of more adequate endoscopic instruments.

Total operating time (OT) was 18 min with an estimated blood loss of 10 ml. No perioperative complications were noted. The time to discharge was 12 hours.

In the subsequent days, she experienced a swift alleviation of symptoms and encountered no complications or symptom recurrence at 3 months follow-up. The patient changed her contraceptive method to combined oral contraceptive pills after this episode.

## Discussion

The unintentional insertion of NuvaRing® in the bladder through the urethra is an extremely rare adverse event reported in the literature (Baker and Barish, 2014; Bhaduri et al., 2009; Ehdaie et al., 2013; Tarragón Gabarró et al., 2009; Teal and Craven, 2006). Patients’ characteristics and diagnostic and therapeutic processes of reported cases are summarized in Table I.

**Table I:** Patients’ characteristics, and diagnostic and therapeutic processes of cases reported in the literature. Abbreviations: US - Ultrasound; CT - Computed tomography.

Authors	Gabarrò et al. (2009)	Ehdaie et al. (2012)	Teal et al. (2006)	Bhaduri et al. (2009)	Baker et al. (2014)
Cases (n)	1 (22 yo)	1 (21 yo)	1 (22 yo)	1 (25 yo)	1 (31 yo)
Comorbidities	No	T7-8 level spinal cord injury at birth is associated with difficulty breech delivery and subsequent neurogenic bladder dysfunction. Previous bladder augmentation surgery and continent urinary diversion using ileum	No	No	No
Symptoms	Dysuria, urinary frequency, haematuria	Suprapubic pain, right hip pain, urgency, vaginal discharge, recurrent urinary tract infections non-responsive to antibiotic therapy	Urinary urgency, frequency, and pelvic pain, which were unresponsive to antibiotic therapy	Persistent low urinary tract symptoms	Urinary frequency, dysuria
Time to diagnosis	Minutes after insertion	8 months	2 months	30 days	3 weeks
Previous antibiotic treatment	Yes (amoxicillin-clavulanate 500mg every 8h, orally – continued for 4 days)	Yes (not specified)	Yes (trimethoprim, sulfamethoxazole, and phenazopyridine)	Yes (trimethoprim, and nitrofurantoin)	Yes (ciprofloxacin)
Diagnostic tools	US + CT +	RX – US + CT +	US – CT +	US + CT +	US not performed. CT +
Cystoscopic removal technique	Cystoscopy with a rigid cystoscope, removed with foreign body forceps.	Cystoscopic extraction with grasping forceps through endoscopic grasping forceps.	Cystoscopic extraction under local anaesthesia.	First attempt (local anaesthesia): failed endoscopic extraction through 23 French cystoscope with 3-prong grasper. Second attempt (general anaesthesia): a thin flexible catheter was used as a lasso to pull the ring out and deliver it through the urethra.	Cystoscopic extraction with a 3-prong grasper (after a failed attempt with alligator grasper).
Post-event contraceptive method	Not specified	The patient switched to depot medroxyprogesterone acetate injections for contraception.	The patient switched to combined oral contraceptive pills.	The patient returned to combined oral contraceptive pills.	The patient switched to combined oral contraceptive pills.

Given the limited number of reported cases, no significant predisposing factors for this event have been identified. Ehdaie et al. (2013) reported a case involving a patient with neurogenic bladder dysfunction, which, while not definitively classifiable as a predisposing factor, did complicate the differential diagnosis process. In all other instances, patients did not present with any comorbidities, including obesity or psychiatric conditions often associated with intravesical foreign bodies, nor did they have a history of urologic or perineal surgeries that could serve as predisposing factors.

All patients reported mild discomfort during the ring insertion followed by the onset of storage and voiding urinary symptoms. The earliest and most reported symptoms were urinary frequency and mild to moderate dysuria. Less frequently, patients complained of urinary urgency, suprapubic pain, and haematuria.

Time from insertion and diagnosis ranged from minutes to eight months, with three patients initially misdiagnosed with urinary tract infections that received ineffective antibiotic treatments. Most patients believed the ring had been unintentionally expelled due to its absence at the scheduled removal time. In 4 out of 5 cases, a pelvic US was performed, effectively identifying intravesical foreign bodies in three cases, whereas in one case it resulted negative due to inadequate bladder fluid filling (Baker and Barish, 2014; Bhaduri et al., 2009; Ehdaie et al., 2013; Tarragón Gabarró et al., 2009; Teal and Craven, 2006). All patients underwent computed tomography (CT) scan readily demonstrating the NuvaRing® within the bladder as a circular hypodense structure (Baker and Barish, 2014; Bhaduri et al., 2009; Tarragón Gabarró et al., 2009; Teal and Craven, 2006). In all instances, the rings were surgically removed by cystoscopy, employing diverse techniques, and encountering varying levels of complexity.

Interestingly, diagnostic cystoscopy showed no urethral or vesical abnormalities in most cases, regardless of the time between symptom onset and diagnosis. The urethra appeared normal, and no bladder mucosal defects, ulcerations, inflammation, or stones were observed. Only Bhaduri et al. (2009) reported a diffuse erythema of the bladder mucosa.

In the report from Tarragón Gabarró et al. (2009), the ring was easily extracted with a single pull utilizing foreign body forceps. Baker and Barish (2014) described a failed attempt with an alligator grasper, which resulted to be too weak to effectively hold the ring, followed by a successful removal through a 3-prong grasper. In the reports from Teal and Craven (2006) and Ehdaie et al. (2013), a single attempt was sufficient for extraction through a non-specified cystoscopic instrument. Bhaduri et al. (2009) described an attempt to retrieve the ring under local anaesthesia, which was aborted due to patient discomfort; a second procedure was performed under general anaesthesia employing a thin flexible catheter as a lasso to pull the ring out through the urethra. No post-operative complications were recorded in any of the reported cases, and all patients experienced a fast resolution of symptoms. In four out of five cases, patients switched to other contraceptive methods (combined oral contraceptive pills or depot medroxyprogesterone acetate injections) (Baker and Barish, 2014; Bhaduri et al., 2009; Ehdaie et al., 2013; Tarragón Gabarró et al., 2009; Teal and Craven, 2006).

Based on our experience, ultrasound serves as a sufficient diagnostic tool for identifying intravesical foreign bodies when performed with an adequately filled bladder, eliminating the need for more invasive and expensive methods such as CT scan. Cystoscopy remains the indispensable procedure for definitive diagnosis and treatment. All diagnostic and therapeutic steps were performed by gynaecologists of our 3rd level Urogynaecology Unit, whereas in a non-tertiary gynaecologic centre without a dedicated unit, a multidisciplinary approach involving both gynaecologists and urologists could be needed to properly evaluate and treat such cases.

To our knowledge, this is the first video to illustrate the retrieval of a misplaced vaginal contraceptive ring from the bladder. Our objective is to present an efficient technique that may be suitable when cystoscopic graspers fail to grasp the ring.

Considering the widespread use of NuvaRing® and similar devices, it is essential to provide comprehensive guidance and instructions regarding its insertion, which should include a demonstrative presentation of the applicator’s functionality.

The rare occurrence of intravesical misplacement must be considered in case of atypical or persistent symptoms in vaginal ring users. Notably, reports of unacknowledged and unintentional ring loss and the emergence of lower urinary tract symptoms should alert healthcare providers to the possibility of this adverse event. Accurate examination is fundamental to prevent misdiagnoses of uncomplicated cystitis or alternative conditions, thus avoiding unnecessary drug administration and diagnostic delays.

## Conclusions

The use of NuvaRing® has substantially increased in recent years due to its efficacy, acceptability, and user satisfaction. The inadvertent placement of NuvaRing® into the bladder is an exceptionally rare complication reported in the literature. This adverse event should always be considered in a NuvaRing® user experiencing low urinary tract symptoms and pelvic pain. Pelvic ultrasound performed in a patient with an adequately fluid-filled bladder is an efficient diagnostic tool for identifying intravesical foreign bodies. More complex methods such as CT scans can be avoided, with cystoscopy remaining the procedure of choice to confirm the diagnosis and complete the treatment. Different cystoscopic approaches have been described for effective removal and our video aims to illustrate a safe and feasible technique, particularly when appropriate cystoscopic graspers are not available. Adequate instruction on NuvaRing® insertion should always be provided, including practical demonstrations, to prevent further cases.

## Video scan (read QR)

https://vimeo.com/928718982/04c518bbb2

**Figure:**